# Book Reviews

**Published:** 1873-10-01

**Authors:** 


### Book Reviews.

Contributions to Practical Surgery. By Geo. W. Norris, M.D., Late Surgeon to Penn. Hospital, etc., etc. Philadelphia: Lindsay & Blakiston. 1873. For sale by Jansen, McClurg & Co., Chicago.

This work is an octavo of 318 pages, and consists of a series of monographs, mainly on fractures, amputation, and the ligation of arteries. The chief value of the work is in the large number of new cases and facts brought out. In arranging these, the writer has produced a number of statistical tables, which will be of great value to the world for a long period.

Surgical statistics are still in their feeble infancy. Many eminent men affect to despise them on account of the worthless character of some of the first efforts in that direction; but if it is true that figures illy digested do often lie, the same is the case with conclusions not based on figures. The truth is this: statistics are no substitute for common reason, nor common reason for statistics. We want the figures to inform us of past facts; and by common sense we decide what bearing those facts have on the present and the future; hence, every collection of surgical facts properly prepared, furnishes food for thought and basis for progress.

But statistics, to be of value, must be properly prepared. Those which our author gives are from the records of Pennsylvania Hospital. If correctly drawn up, that is, if they honestly represent all the cases of the kinds tabulated occurring in the Hospital, they are of great and permanent value, for they are surgical facts, fairly stated; but the same cannot be said of the tables collated from cases found scattered in medical books and journals. There is a fallacy here. On the average, a man who has ligated a carotid artery, for instance, and cured the patient, feels much more like publishing the case than one who has done the same operation and lost a life. The latter feels depressed and gloomy, and, as a general rule, not inclined to print his disaster; hence the journals contain a preponderance of the successful cases. Now, if one looks over the files, and gathers into a table these scattered operations, he will get far too much rose-color in his picture. The fatal cases are not visible; and his table is almost utterly worthless for determining the mortality. One imperative law of useful statistics is this: The facts taken for tabulation must be in groups which contain the success and failures in impartial proportion.

On the whole, Dr. Norris' book is a decidedly valuable contribution to our literature, and will be useful for a long time to come. E. A.

Chemical Electro-Therapeutics, Medical and Surgical; a Handbook for Physicians in the Treatment of Nervous and other Diseases. By Allan McLane Hamilton, M.D. New York: D. Appleton & Co. W. B. Keen & Cooke, Chicago.

In this little volume of 187 pages the author claims merely to have formed a compilation of well-tried measures and reported cases, without attempting to enter into any detailed descriptions of the diseases alluded to. The first third of the work is devoted to a general consideration of Electrical phenomena and Electro-Therapeutics. In the next three chapters, under the heading of Special Electro-Therapeutics, the application of electricity in the different varieties of paralysis hyperæsthesia convulsive diseases, etc., are considered. Chapters on Electrolysis and Galvano-Causty, with an appendix on the management of Batteries, close the volume.

The Cerebral Convolutions of Man, represented according to original observations, especially upon the Development of the Fœtus. Intended for the use of Physicians. By Alexander Ecker, Professor of Anatomy at the University of Freiburg, Baden. Translated by Robert T. Edes, M.D. New-York: D. Appleton & Co. Jansen, McClurg & Co., Chicago. 8vo., 86 pp.

